# Satellite Glial Cells in Peripheral Nerve Injury and Regeneration

**DOI:** 10.3390/biomedicines14030660

**Published:** 2026-03-13

**Authors:** Linjia Hu, Haimin Lu, Yufan Shen, Zige Peng, Yinying Shen, Qiong Cheng, Yang Gu

**Affiliations:** Nantong University Xinlin College, Jiangsu Key Laboratory of Tissue Engineering and Neuroregeneration, Key Laboratory of Neuroregeneration of Ministry of Education, Co-Innovation Center of Neuroregeneration, Nantong University, Nantong 226001, China; hlj12983216371127@163.com (L.H.); 2424310004@stmail.ntu.edu.cn (H.L.); 15862474245@163.com (Y.S.); pengzige301@126.com (Z.P.)

**Keywords:** satellite glial cells, peripheral nervous system, neuron, peripheral nerve injury, axonal regeneration

## Abstract

Satellite glial cells (SGCs) are morphologically unique peripheral glial cells that surround neuronal somas in sensory, sympathetic, and parasympathetic ganglia. Satellite glial cells communicate with neurons that they ensheathe and form a distinct structural and functional unit. Following peripheral nerve injury, satellite glial cells undergo remarkable morphological changes, including gliosis, and help regulate the microenvironment surrounding neuronal somas. The expression of many satellite glial cell markers such as glial fibrillary acidic protein (GFAP) and connexin-43, pro-inflammatory cytokines, and growth factors in satellite glial cells is altered in these cells. Injury responses of satellite glial cells, particularly the activation of peroxisome proliferator-activated receptor α (PPARα), contribute to enhanced axonal regeneration. Targeting satellite glial cells may therefore offer novel therapeutic strategies for the treatment of peripheral nerve injury.

## 1. Introduction

Glial cells are supportive non-neuronal cells in the entire nervous system. Most studies on glial cells have focused on microglia, oligodendrocytes, astrocytes, and retinal Müller cells in the central nervous system as well as Schwann cells in the peripheral nervous system. A growing number of studies have demonstrated that besides myelin-forming Schwann cells, satellite glial cells (SGCs) represent an important glial cell population in the peripheral nervous system.

Satellite glial cells are homeostatic cells located in sensory, sympathetic, and parasympathetic ganglia. They enwrap around neuronal somas, form a functional unit with neurons, and participate in the regulation of neuronal behavior under physiological and various pathological conditions [1]. The aim of the present review is to describe the normal features of satellite glial cells and their changes following peripheral nerve injury, a common neurological disorder. The functional roles of satellite glial cells in the elongation and regeneration of injured axons and the functional recovery of injured nerves are summarized, aiming to reveal the potential applications of satellite glial cells in the treatment of peripheral nerve injury.

## 2. Characteristics of Satellite Glial Cells

### 2.1. Morphological Characteristics of Satellite Glial Cells

**Figure 1:**
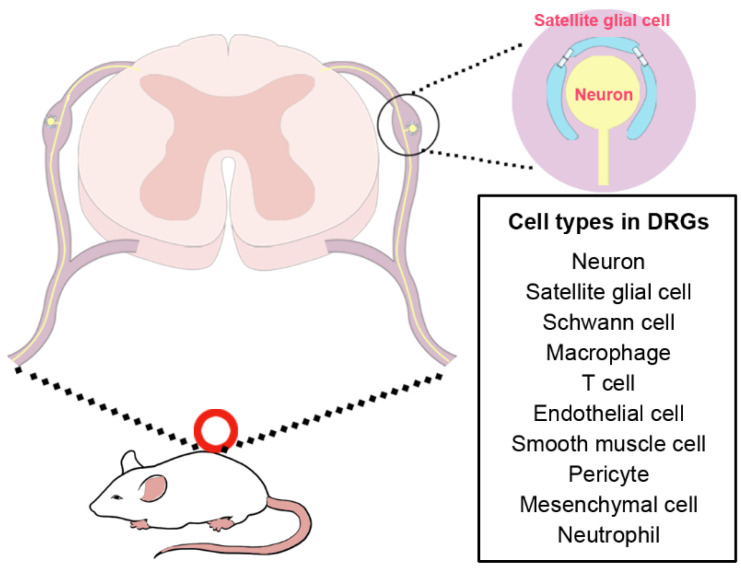
Anatomical structure of satellite glial cells and the cell types in the dorsal root ganglion.

Satellite glial cells in peripheral ganglia are morphologically distinguishable as they closely surround and envelop neuronal cell bodies. These cells are flat in shape, with small volume but large surface to volume ratio [2]. They intermingle with the processes of adjacent satellite glial cells, connect with adjacent satellite glial cells via gap junctions, and jointly wrap neuronal somas. Satellite glial cells exhibit morphological plasticity, with the number of SGCs enveloping a neuronal soma being positively correlated with neuronal volume. Interestingly, this enveloping ratio varies across species and is positively correlated with neuronal volume. For instance, in the thoracic spinal ganglia of lizards or geckos, the mean neuronal soma volume is approximately 6500 μm^3^, which is associated with an average of 3.8 SGCs per neuron. This number is significantly higher in mammals: in mice, the mean neuronal volume is about 9100 μm^3^, with an average of 5.5 SGCs per neuron; this ratio increases to 8.2 in rats and 10.2 in rabbits, respectively [3]. In occasional cases (9.4% in lizards and 5.6% in rats), two or three neuronal somas are grouped together and enveloped by a common sheath of SGCs [4]. Unlike sensory neurons, neurons in sympathetic and parasympathetic ganglia receive synapses; therefore, SGCs in autonomic ganglia not only envelop neuronal somas but also enclose and cover neuronal processes [5]. The structural features and cellular composition discussed above are schematically represented in Figure 1, which illustrates the anatomical organization of satellite glial cells within the dorsal root ganglion (DRG).

The envelopment of satellite glial cells around the surface areas of neurons in peripheral ganglia isolates individual neurons from adjacent neurons and effectively reduces the diffusion of substances, especially large molecules, circulating in the blood towards the extracellular space around neurons. Although satellite glial cells regulate the neuronal microenvironment, they are only occasionally linked by tight junctions and are not capable of forming a tight barrier similar to the blood–brain barrier. The envelopment of satellite glial cells on the neuronal surface may not be continuous, leaving occasional gaps that permit extracellular exchange [5]. To assess the permeability of this envelope at the ultrastructural level, two studies using tracer-based observations have been reported. It was observed that horseradish peroxidase injected into blood vessels can penetrate into the perineuronal spaces in the DRG [6]. Furthermore, using lanthanum nitrate as an electron-dense tracer, it was demonstrated that lanthanum ions can diffuse into the narrow cleft between satellite glial cells and neurons through the seams between adjacent satellite glial cells [7]. These findings provide direct evidence that extracellular substances can penetrate the satellite glial cell envelope, confirming that the sheath acts as a selective regulator rather than a complete barrier. However, the permeability of the satellite glial cell envelope appears to vary across different types of peripheral ganglia. A comparative study indicates that, compared with sensory ganglia, a more effective barrier exists in autonomic ganglia: injected wheat germ agglutinin-horseradish peroxidase and horseradish peroxidase were detected in the space between satellite glial cells and neurons in the trigeminal ganglion but were absent in the extracellular space of neurons in the superior cervical ganglion [8]. Whether in the tightly arranged structure of autonomic ganglia or the relatively loosely arranged structure of sensory ganglia, the envelopment of satellite glial cells around neurons forms a morphological and functional unit and facilitates communication between satellite glial cells and neurons. In the satellite glial cell–neuron structural unit, satellite glial cells are in close contact with the neuronal soma and the distance between satellite glial cells and neuronal surface is only about 20 μm [9]. In addition, sensory neurons extend slender projections that are similar to microvilli toward satellite glial cells [10]. This further narrows the distance between satellite glial cells and neuronal soma and facilitates chemical and metabolic exchange.

### 2.2. Molecular Characteristics of Satellite Glial Cells

The presence of molecular markers of satellite glial cells, together with the unique ring structure around the neuronal soma, enables the identification of satellite glial cells in the peripheral ganglion. Similar to other types of glial cells such as astrocytes and Schwann cells, satellite glial cells express S100B calcium-binding protein and glial fibrillary acidic protein (GFAP), although the expression of GFAP in satellite glial cells is much lower than that in astrocytes under homeostatic conditions. Glutamine synthetase (GS), an integral enzyme of the glutamine cycle that mediates adenosine triphosphate-dependent amidation of glutamate to glutamine, is a more specific cellular marker of satellite glial cells, although it is also expressed in Schwann cells [11]. The high abundance of glutamine synthetase in satellite glial cells helps the removal of glutamate in the extracellular space between satellite glial cells and neurons and prevents cytotoxicity induced by excess glutamate [12]. The observations that other molecules responsible for glutamate uptake, such as glutamate-aspartate transporter and glial glutamate transporter (GLAST), are also expressed in satellite glial cells further demonstrate the regulatory roles of satellite glial cells in glutamate homeostasis [13]. A recent study, by adding exogenous L-glutamine, directly demonstrates that glutamate supports the proliferation and inhibits the apoptosis of satellite glial cells, indicating the involvement of glutamatergic metabolism in modulating satellite glial cell activity [14].

Potassium channels, especially inward rectifying potassium channel Kir4.1 and small-conductance calcium-activated potassium channel 3 (SK3), have been found to be expressed in satellite glial cells but are absent in neurons and are considered satellite glial cell markers [15,16]. The gap junction protein connexin-43, a molecule involved in potassium ion buffering, is co-localized with glutamine synthetase in perineuronal satellite glial cells [16,17]. The cell–cell adhesion molecule cadherin 19, a glycoprotein previously identified as the only unique marker for Schwann cell precursors, is expressed in satellite glial cells in both embryonic and adult rat cervical DRG. The presence of cadherin 19 in satellite glial cells demonstrates that although they are generally considered to be analogous to astrocytes, satellite glial cells share certain features with Schwann cells, especially Schwann cell precursors [18]. The observation that the expression of cadherin 19 rapidly diminishes after loss of neuronal contact implies that cadherin 19 may be involved in maintaining the cellular interactions between satellite glial cells and neurons [18].

**Figure 2:**
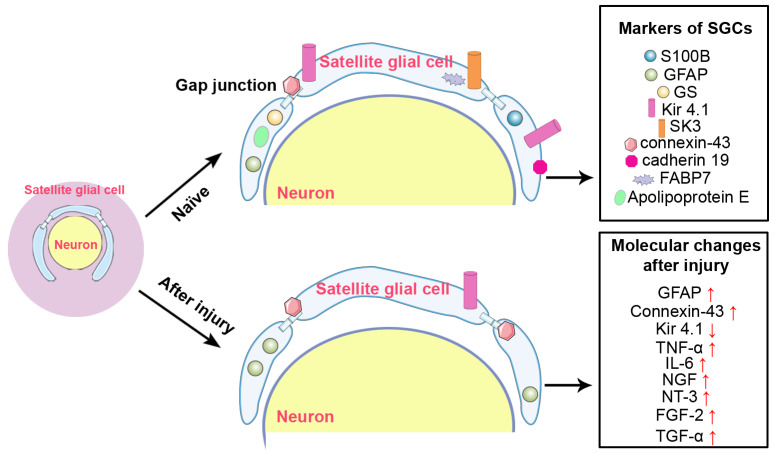
The molecular markers of satellite glial cells and molecular changes in SGCs following peripheral nerve injury. Naïve state: The molecules shown in this panel are constitutively expressed in SGCs under naïve conditions and serve as specific markers to identify SGCs, distinguishing them from neurons and other cell types within the dorsal root ganglia. GFAP: glial fibrillary acidic protein; GS: glutamine synthetase; SK3: small-conductance calcium-activated potassium channel 3; FABP7: fatty acid binding protein 7. Injury state: The molecules shown in this panel are those reported in the articles to exhibit altered expression following injury. They include both SGC markers and non-marker molecules that are upregulated in response to injury. GFAP: glial fibrillary acidic protein; TNF-α: tumor necrosis factor α; IL-6: interleukin-6; NGF: nerve growth factor; NT-3: neurotrophin-3; FGF-2: fibroblast growth factor-2; TGF-α: transforming growth factor α. ↑: upregulation; ↓: downregulation.

The application of single cell sequencing to the peripheral nervous system greatly facilitates the identification of specific cellular markers. Single cell profiling of mouse DRG reveals the high abundance of fatty acid binding protein 7 (FABP7), which is a nervous system specific protein that binds long-chain fatty acids and is involved in the uptake and transport of fatty acids, in satellite glial cells [19]. In addition to sensory ganglia, FABP7 and S100B are also recognized as specific markers for satellite glial cells in mouse stellate ganglia, which are sympathetic ganglia [20]. Another study examined the expression levels of FABP7 as well as other satellite glial cell markers including S100B, Kir4.1 and SK3 in sympathetic ganglia of rat at different developmental stages and demonstrates that, different from sensory ganglia, the expression of FABP7 is relatively low in adult sympathetic ganglia. FABP7 expression is reduced during postnatal development, while S100B, Kir4.1 and SK3 undergo opposite age-dependent changes and are highly expressed in adult sympathetic satellite glial cells [21]. Besides FABP7, apolipoprotein E, another protein involved in lipid transport and metabolism, is also considered as a specific marker that distinguishes satellite glial cells from other cell populations, implying that satellite glial cells may be closely associated with fatty acid signaling [22,23]. The molecular markers of satellite glial cells are summarized in Figure 2.

### 2.3. Communication Between Satellite Glial Cells and Neurons

Satellite glial cells interact with each other via gap junctions. The presence of gap junctions connects the cytoplasm of adjacent satellite glial cells, facilitates direct exchange of ions and small molecules, and mediates fast intercellular communication. The communication between satellite glial cells and the neurons that they ensheathe is more complicated. Purinergic signaling plays essential roles in cell–cell communication in peripheral ganglion. Neuronal somas, when electrically or chemically stimulated, release neuroglial transmitters such as ATP and glutamate. In DRG neurons, it is observed that after the electrical stimulation of sensory neurons and influx of external calcium, vesicular ATP is released from the neuronal soma. ATP activates ionotropic purinergic P2X7 receptors in satellite glial cells and induces upregulated production and secretion of the cytokine tumor necrosis factor α (TNF-α). Elevated levels of TNF-α released from satellite glial cells enhance P2X3 receptor-mediated currents in neurons and increase neuronal excitability [24,25]. Application of α,β-methylene ATP, a phosphonic analog of ATP, to mouse DRG quickly activates P2X3 receptors that are present in high levels in small sensory neurons (neurons with soma areas less than 450 μm^2^) and induces the opening of pannexin 1 channel and the subsequent release of ATP. ATP released from small sensory neurons activates large sensory neurons through P2X4 receptors and the relatively small amount of P2X3 receptors in large neurons (neurons with soma areas greater than 700 μm^2^) and activates satellite glial cells through P2X7 receptors and G-protein coupled metabotropic P2Y receptors. Activated large sensory neurons and satellite glial cells also release ATP to the extracellular space [26]. Emerging studies demonstrate that not only neurons with different sizes have distinct expressions of purinergic receptors, but satellite glial cells also exhibit considerable heterogeneity with diverse expressions of genes associated with purinergic signaling [27]. For instance, a subtype of satellite glial cells expresses TRP vanilloid 4 and have stronger P2Y1 receptor-mediated responses via P2Y1 receptor-TRP vanilloid 4 coupling [28]. In addition to the excitatory effects of satellite glial cells on the neurons they envelop, satellite glial cells may also exert inhibitory actions via cross-talk between P2X7, P2Y1, and P2X3 receptors. Activated P2X7 receptors in satellite glial cells stimulate the release of ATP from these cells. This released ATP, by activating P2Y1 receptors in neurons, reduces P2X3 receptor expression and decreases neuronal activity [25,29].

Similar to purinergic receptors, functional glutamate receptors, such as N-methyl-D-aspartate (NMDA) receptors, have been found to be expressed in satellite glial cells and neurons in sensory ganglion [30,31]. Stimulation of sensory neurons via KCl depolarization increases glutamate release from neurons and thus evokes calcium influx in both satellite glial cells and neurons [32]. The presence of NMDA receptor in satellite glial cells, together with the finding that DRG neurons can release L-aspartate, suggests that besides glutamate, other excitatory amino acids such as L-aspartate may mediate the communication between satellite glial cells and neurons [33]. In addition, except for TNF-α, satellite glial cells can secrete a variety of bioactive molecules such as nerve growth factor (NGF), interleukin-6 (IL-6), and interleukin-1β [34,35,36]. Intercellular exchange of these bioactive molecules may facilitate bidirectional communication between satellite glial cells and neurons and contribute to the processing of afferent information. Under certain circumstances, such as traumatic injury, inflammation, and virus infection, changes of cellular communication in peripheral ganglia contribute to neuropathological changes.

## 3. Responses of Satellite Glial Cells to Peripheral Nerve Injury

Peripheral nerve injury leads to structural disruptions, induces partial or complete functional loss, and severely impairs patients’ quality of life. Following peripheral nerve injury, the damaged neuronal axon transmits injury signals to the neuronal soma via electrical conduction and/or axonal transport, which elicits the synthesis and secretion of chemical messengers such as nitric oxide and ATP from the neuronal soma, influences surrounding satellite glial cells via cell–cell interactions, and triggers injury responses in satellite glial cells [37].

### 3.1. Morphological Changes of Satellite Glial Cells Following Peripheral Nerve Injury

Satellite glial cells are dynamic cells and undergo gliosis in response to injury signals. Immunohistochemical analyses show that in rat superior cervical ganglion, not only the number of neurons but also the number of satellite glial cells decreases at 12 and 18 h after the transection of the internal and external carotid nerves, indicating that, similar to neurons, satellite glial cells also react to axonal injury with apoptosis [38]. Unlike neurons, which generally do not retain the ability to proliferate, satellite glial cells are capable of self-renewal and can proliferate to replace lost cell populations. The number of neurons decreases to 10% of the original number in the superior cervical ganglion of 6-day-old rats with no recovery at 28 postpartum, while the number of non-neuronal cells first decreases to 70% and then recovers to the normal level [39]. These non-neuronal cells with proliferative ability after nerve injury are presumed to be satellite glial cells. Immunostaining of injured peripheral ganglia with satellite glial cell markers confirms this hypothesis and demonstrates the replenishment of satellite glial cells. The number of GFAP-positive satellite glial cells increases in both the maxillary and mandibular nerve regions at 7 days after the extraction of rat upper molars [40]. 5-Bromo-2′-deoxyuridine (BrdU) and SK3 double-positive satellite glial cells, indicating active proliferation, are observed in rat trigeminal ganglion from 1 day after chronic constriction injury, reaching a proliferation peak at 4 days post-injury [41]. In addition to changes in cell number, following nerve injury, the number of satellite glial cell layers around the neurons is also altered. Examination of the fine structure of satellite glial cells in rabbit superior cervical ganglia shows that at 1 to 3 weeks after postganglionic axotomy, the number of satellite glial cell layers around neurons decreases from up to 10 overlapping layers to only 1 to 2 layers. This may occur as satellite glial cells slide over one another to accommodate the doubling of neuronal size during chromatolysis and more effectively package the enlarged neuronal soma [5,42]. However, it is not clear how the increased number of satellite glial cells are arranged to enwrap neurons in cases of severe peripheral nerve injury in which a large number of neurons die. This decreased number of satellite glial cell layers around neurons may be associated with reduced barrier function and hence immune cells that are initially located outside the satellite glial cell layers have been detected infiltrating the satellite glial cell–neuron unit and positioning themselves against neuronal somas [43,44].

A recent study, using flow cytometric analysis of cells in dissected DRG from mice that underwent partial sciatic nerve ligation, presents findings that contrast with earlier reports and suggests that satellite glial cells do not proliferate after nerve injury, or that their proliferation rate of satellite glial cells is far less than macrophages. Consistent with this, sequencing data of isolated and sorted satellite glial cells in injured mice DRG did not reveal any significant changes in proliferation-associated genes [44]. Single cell transcriptomic profiling of naïve mouse DRG has shown the presence of the proliferation marker Mki67 in a sub-population of satellite glial cells enriched for extracellular matrix and cell adhesion pathways [45]. However, after nerve injury, the percentage of this satellite glial cell sub-population in total satellite glial cells does not appear to increase [45]. Mki67 as well as another cell cycle marker Cdk1 was found to be expressed in macrophages but not in satellite glial cells under injured conditions [19]. These findings raise the possibility that previously reported proliferating satellite glial cells in injured peripheral ganglia may actually be infiltrating macrophages [44]. The observation that a subset of cells co-expressing markers of both satellite glial cells and macrophages emerges after nerve injury adds to the complexity of cell type identification [45]. To determine whether satellite glial cells proliferate in response to axonal injuries, further studies are needed to more clearly distinguish satellite glial cells from macrophages and to investigate the cellular states of satellite glial cells.

Moreover, satellite glial cells share certain characteristics with macrophages in that they have immune properties and express several markers also found in macrophages, such as CD14, CD68, and CD11b. Satellite glial cells also express major histocompatibility complex class II (MHC class II) and are thus considered tissue-resident antigen-presenting cells in peripheral ganglion [46]. Following nerve injury, the expression of MHC class II in satellite glial cells is markedly elevated and the ClueGO term MHC protein complex is significantly enriched among differentially expressed genes in satellite glial cells, suggesting that they may exert immune-related functions in response to injury signals [44]. Ultrastructural observations of injured peripheral ganglia show that satellite glial cells undergo remarkable fine structural changes, including the appearance of cytoplasmic endocytotic vesicles and an increase in filaments in the early stage after nerve injury, as well as the enlargement of mitochondria at a relatively later stage [5,42]. The presence of endocytotic vesicles in the satellite glial cell cytoplasm indicates that, similar to macrophages and numerous glial cells, such as microglia, astrocytes, and Schwann cells, satellite glial cells process phagocytic activity and are capable of engulfing and clearing neuronal debris. By removing injury-induced, neuron-derived debris, satellite glial cells help maintain nerve homeostasis and remodel the microenvironment in injured peripheral ganglia.

### 3.2. Molecular Changes in Satellite Glial Cells Following Peripheral Nerve Injury

In addition to changes in the morphology of satellite glial cells, many satellite glial cell markers exhibit different expression patterns after axonal injury. Increased immunofluorescence intensity for GFAP was detected in rat DRG by 3 days after sciatic nerve transection and persisted until 6 weeks after nerve injury. Furthermore, compared with the DRG at the uninjured side where the only approximately 15% of neuronal somas are surrounded by GFAP-positive satellite glial cells, the portion of neuronal somas surrounded by GFAP-positive satellite glial cells increased nearly six-fold [47]. An increased level of GFAP was also observed in rat trigeminal ganglia after chronic constriction injury of the infraorbital nerve, rat trigeminal ganglia after dental injury, as well as mouse DRG after spared nerve injury, especially in satellite glial cells around activating transcription factor 3 (ATF-3)-positive neurons [41,48,49]. Elevated GFAP expression in satellite glial cells is thus considered as a marker of satellite glial cell activation. Functionally, increased amount of GFAP may facilitate the clearance of cytotoxic levels of accumulated glutamate, as GFAP interacts with the GLAST, which can take up glutamate [12,50]. However, a comparative analysis indicates that the upregulation of GFAP in satellite glial cells is species and disease-dependent. GFAP upregulation in satellite glial cells is more pronounced in a systemic inflammation model than in an injury model and is also more pronounced in rats after sciatic nerve ligation compared with mouse [51].

Besides GFAP, another satellite glial cell marker, which is the connexin-43 gap-junction subunit, has also been found to be upregulated in the satellite glial cells after nerve injury. In rat trigeminal ganglia ipsilateral to chronic constriction injury of the infraorbital nerve, the protein level of connexin-43 increases significantly at 10 days post-injury, while its expression in the contralateral trigeminal ganglia remains unaltered [52]. The expression of connexin-43 in satellite glial cells in rat trigeminal ganglia increases by more than two-fold on day 8 after inferior alveolar nerve transection-induced mandibular nerve injury and gradually returns toward the normal level at later time points [53]. Consistent with the elevated expression of the gap junction protein connexin-43, enhanced intercellular communication between adjacent satellite glial cells is observed in injured peripheral ganglia, and the occurrence of coupling between satellite glial cells surrounding different neurons increases substantially, indicating that activated satellite glial cells after nerve injury participate in bridging separate perineuronal sheaths [5].

Unlike GFAP and connexin-43, the potassium channel Kir4.1 is markedly suppressed after nerve injury. Western blot analysis demonstrates that the protein level of Kir4.1 decreases by about 40% in the trigeminal ganglion of rats subjected to a 10-day chronic constriction injury of the infraorbital nerve [15]. Gene expression profiling of satellite glial cells in the DRG shows that in mouse DRG, the expression of the Kir4.1-encoding gene *Kcnj10* in satellite glial cells decreases after sciatic nerve axotomy [23]. Patch clamp recording reveals that satellite glial cells in the chronically compressed DRG exhibit increased input resistance and diminished inward and outward currents due to inactivated potassium channels [54]. Reduced expression of Kir4.1 leads to decreased inwardly rectifying potassium currents in satellite glial cells, resulting in the accumulation of extracellular potassium and increased neuronal excitability.

In addition to changes in satellite glial cell markers, the production and secretion of many pro-inflammatory cytokines are markedly increased in activated satellite glial cells [55]. The expression of TNF-α in satellite glial cells has been shown to be elevated in the DRG in numerous injury models, such as ventral root transection and sciatic nerve crush [56,57]. IL-6 levels are increased in satellite glial cells in rat DRG after chronic constriction injury of the sciatic nerve, and IL-6 mRNA and protein levels are upregulated in both the ipsilateral and contralateral sides after unilateral chronic constriction injury compared with naïve rats [58,59]. Elevated IL-6 in satellite glial cells leads to the phosphorylation of signal transducer and activator of transcription 3 (STAT3) and subsequent activation of STAT3 signaling [58].

Satellite glial cells also synthesize and secrete a variety of growth factors, including neurotrophic factors. NGF and neurotrophin-3 (NT-3) are expressed at low levels in rat DRG at the naïve state. As early as 2 days after sciatic nerve transection, the expression of NGF and NT-3 in satellite glial cells has been shown to increase. Unlike IL-6, NGF and NT-3 are only upregulated in the ipsilateral, not contralateral, DRG after sciatic nerve injury [60]. Fibroblast growth factor-2 (FGF-2) has been observed to be markedly upregulated at an early time point after nerve injury in the DRG of rats that underwent sciatic nerve crush and in the superior cervical ganglion of rats that underwent carotid nerve transection [61,62,63]. Transforming growth factor α (TGF-α) is elevated in rat ipsilateral and contralateral DRG after sciatic nerve transection, and satellite glial cells are the main source of the elevated TGF-α [64]. Following axotomy, the production and secretion by satellite glial cells of neurotrophins and cytokines with pro-regenerative roles suggests that satellite glial cells may directly communicate with the neuronal somas that they ensheath during the regeneration process. The intercellular trophic interactions between satellite glial cells and neuronal somas may be of great significance, as nerve injury disrupts axons and prevents the supply of bioactive molecules essential for regeneration from the periphery. The molecular changes in satellite glial cells following peripheral nerve injury are summarized in Figure 2. In addition to this schematic overview, the expression changes of these molecules and their functional consequences are comprehensively summarized in Table 1.

### 3.3. Satellite Glial Cells in Nerve Regeneration

Remarkable molecular changes in satellite glial cells following nerve injury, such as elevated connexin-43 expression and reduced Kir4.1 expression, are associated with neuropathic pain [65]. Alterations in purinergic signaling after axotomy, a key signaling pathway that mediates satellite glial cell and neuron interaction, induce the secretion of inflammatory cytokines and contribute to the regulation of neuropathic pain [66]. Many of these dysregulated factors, for example, IL-6, are also closely associated with axonal regeneration [67]. Hence, beyond elucidating the essential roles of satellite glial cells in neuropathic pain, their functional involvement in nerve regeneration also warrants investigation.

Consistent with the high abundance of fatty acid-associated molecules, such as FABP7, in satellite glial cells, lipid-related biological activities are implicated in the regeneration process. Differentially expressed genes in isolated satellite glial cells in mouse DRG at both 3 days and 14 days after sciatic nerve ligation are enriched in the ClueGO term cholesterol biosynthesis [44]. Single cell sequencing data of mouse DRG at 3 days after sciatic nerve crush also reveals significant enrichment of the lipid-related pathway fatty acid metabolism in differentially expressed genes in the satellite glial cell cluster [19]. Conditional deletion of fatty acid synthase (FASN), a key regulator of the committed step in endogenous fatty acid synthesis, suppresses the elevation of ATF3 expression, hinders neurite growth and elongation, and impairs nerve regeneration. In contrast, activating peroxisome proliferator-activated receptor α (PPARα), a transcription factor that regulates genes in lipid metabolism, in satellite glial cells promotes neurite growth in a satellite glial cell–neuron co-culture system and accelerates the regeneration of injured axons after sciatic nerve crush [19].

It is worth noting that DRG neurons are pseudounipolar neurons with peripheral axonal branches that innervate the periphery and central axonal branches that transmit sensory signals to neurons in the spinal cord. The regeneration capacity of sensory neurons after injury to the central axonal branches is considerably lower than that of peripheral axonal branches [68]. Nevertheless, similar to the promoting role of activated PPARα signaling in the regeneration of injured sciatic nerves, the application of a PPARα agonist is capable of enhancing axon elongation after a crush injury of the central axonal branch in the dorsal column [45]. The fact that the PPAR signaling pathway is only enriched in upregulated genes in the satellite glial cells after sciatic nerve crush injury, but not significantly enriched in differentially expressed genes after dorsal root crush or spinal cord injury, suggests that the lack of PPARα activation in satellite glial cells after central axonal branch injury may be one of the mechanisms underlying the poor regenerative ability of injured central axonal branches. The identification of a unique satellite glial cell subpopulation in the DRG only after sciatic nerve crush, and not after dorsal root crush or spinal cord injury, further suggests that robust injury responses of satellite glial cells contribute to successful regeneration [45].

In contrast, satellite glial cells may play inhibitory roles in axonal regeneration as laser ablation of satellite glial cells in larval zebrafish accelerates neurite growth and promotes the extension of injured central axons into the spinal cord [69]. In addition, it is demonstrated that under chronic psychological stress, reduced Kir4.1 expression in mouse satellite glial cells in the DRG increases the concentration of extracellular potassium, which in turn depolarizes neurons and induces neuronal hyperactivation, causes reactive oxygen species accumulation and mitochondrial damage, and impairs the regrowth of central axonal branches after preconditioning peripheral nerve lesion [70]. Given that the abundance of Kir4.1 in satellite glial cells is generally reduced after axotomy, satellite glial cells may exert negative effects on axonal regeneration. However, increased reactive oxygen species can stimulate the regeneration of peripheral axonal branches by activating the phosphatidylinositol 3-kinase (PI3K)-phosphorylated Akt (p-Akt) signaling pathway [71]. These findings underscore the functional complexity of satellite glial cells and highlight the importance of crosstalk between satellite glial cells and neurons during nerve injury and regeneration. The dual roles of satellite glial cells in nerve regeneration are systematically summarized in Table 2.

## 4. Conclusions and Perspectives

Satellite glial cells are a major and important cell population in sensory, sympathetic, and parasympathetic ganglia. Satellite glial cells surround neuronal somas, communicate with neurons, and participate in the regulation of various biological activities, including the regeneration of injured axons. Following peripheral nerve injury, satellite glial cells undergo morphological and molecular alterations, express numerous cytokines and growth factors, and contribute to axonal elongation and nerve regeneration by modulating the microenvironment around neurons. Neurons have distinct subtypes with unique transcriptional features and different injury responses [22]. The fact that GFAP is particularly highly expressed in satellite glial cells surrounding ATF-3-postive neurons implies that satellite glial cells surrounding different neuronal subtypes may respond differentially to injury signals [41]. Consequently, it is of great interest to explore and compare the injury responses of those surrounding diverse neuronal sub-types. Communications between satellite glial cells and other cell types in peripheral ganglia, apart from neurons, such as Schwann cells, fibroblasts, and macrophages, also warrants investigation. Lastly, we have focused on the functional roles of satellite glial cells in axonal elongation after peripheral nerve injury, since injury to peripheral axonal branches generally does not lead to significant neuronal loss [72]. However, under severe injury conditions, such as spinal cord injury that affects the central axonal branches of DRG, neuronal death may occur. The role of satellite glial cells in regulating neuronal apoptosis and survival remains to be elucidated in future studies.

## Figures and Tables

**Table 1:** Summary of molecule expression changes and functional roles of SGCs following nerve injury.

Molecule	Injury Model	Function
GFAP	-chronic constriction injury of the infraorbital nerve -sciatic nerve transection -dental injury	marks SGC activation and phenotypic switch from resting to activated state
connexin-43	-chronic constriction injury of the infraorbital nerve -inferior alveolar nerve injury	increases gap junction coupling between SGCs
Kir4.1	chronic constriction injury of the infraorbital nerve	reduces SGC’s K+ buffering capacity, leading to elevated extracellular K+
TNF-α	-Ventral root transection -sciatic nerve crush	acts in a paracrine manner on neuronal TNFR1 to enhance excitability
IL-6	chronic constriction injury of the sciatic nerve	establishes an autocrine/paracrine loop, activates STAT3 pathway
NGF	sciatic nerve transection/crush	drives sympathetic sprouting, contributing to sympathetically maintained pain
NT-3	sciatic nerve transection/crush	synergizes with NGF to induce sympathetic sprouting
FGF-2	-sciatic nerve crush -superior cervical ganglion axotomy	acts as an autocrine/paracrine mitogen, driving SGC proliferation
TGF-α	sciatic nerve transection	orchestrates neuronal support and glial activation/proliferation

TNFR1: tumor necrosis factor receptor 1.

**Table 2:** Roles of satellite glial cells in nerve regeneration.

Role	Key Molecules/Pathways	Function	References
promote nerve regeneration	-FABP7, fatty acid metabolism -cholesterol synthesis	-provide membrane precursors and energy substrates for regeneration -facilitate the interaction between SGC neurons	[19,44]
-PPARα, PPARα agonist -FASN, fatty acid synthesis		-promote the growth and extension of nerve fibers -accelerate the regeneration of damaged axons	[19,45]
unique SGC subpopulation (appears only after injury to the peripheral branch)		provide sufficient damage response to promote successful regeneration	[45]
IL-6		support axonal regeneration	[67]
inhibit nerve regeneration SGC ablation experiment (Zebrafish larval model)		accelerate the growth of nerve fibers	[69]
Kir4.1		inhibit central axon regeneration	[70]
dual roles	ROS	-inhibit the regeneration of the central branches -promote peripheral branch regeneration through Pl3K/Akt signaling pathway	[71]

## Data Availability

No new data were created or analyzed in this study. Data sharing is not applicable to this article.

## Abbreviations

ATF3	activating transcription factor 3
BrdU	5-bromo-2′-deoxyuridine
DRG	dorsal root ganglion
FABP7	fatty acid binding protein 7
FASN	fatty acid synthase
FGF2	fibroblast growth factor-2
GFAP	glial fibrillary acidic protein
GLAST	glutamate transporter
GS	glutamine synthetase
IL-6	interleukin-6
MHC class II	major histocompatibility complex class II
NGF	nerve growth factor
NMDA	N-methyl-D-aspartate
NT-3	neurotrophin-3
PI3K	phosphatidylinositol 3-kinase
p-Akt	phosphorylated Akt
PPARα	peroxisome proliferator-activated receptor α
SGC	satellite glial cell
SK3	small-conductance calcium-activated potassium channel 3
STAT3	signal transducer and activator of transcription 3
TGF-α	transforming growth factor α
TNF-α	tumor necrosis factor α
TNFR1	tumor necrosis factor receptor 1

## References

[1] Hanani M., Spray D.C. (2020). Emerging Importance of Satellite Glia in Nervous System Function and Dysfunction. Nat. Rev. Neurosci..

[2] Hanani M., Verkhratsky A. (2021). Satellite Glial Cells and Astrocytes, a Comparative Review. Neurochem. Res..

[3] Ledda M., De Palo S., Pannese E. (2004). Ratios between Number of Neuroglial Cells and Number and Volume of Nerve Cells in the Spinal Ganglia of Two Species of Reptiles and Three Species of Mammals. Tissue Cell.

[4] Pannese E., Ledda M., Arcidiacono G., Rigamonti L. (1991). Clusters of Nerve Cell Bodies Enclosed within a Common Connective Tissue Envelope in the Spinal Ganglia of the Lizard and Rat. Cell Tissue Res..

[5] Hanani M. (2010). Satellite Glial Cells in Sympathetic and Parasympathetic Ganglia: In Search of Function. Brain Res. Rev..

[6] Jacobs J.M., Macfarlane R.M., Cavanagh J.B. (1976). Vascular Leakage in the Dorsal Root Ganglia of the Rat, Studied with Horseradish Peroxidase. J. Neurol. Sci..

[7] Shinder V., Devor M. (1994). Structural Basis of Neuron-to-Neuron Cross-Excitation in Dorsal Root Ganglia. J. Neurocytol..

[8] Ten Tusscher M.P., Klooster J., Vrensen G.F. (1989). Satellite Cells as Blood-Ganglion Cell Barrier in Autonomic Ganglia. Brain Res..

[9] Pannese E. (1981). The Satellite Cells of the Sensory Ganglia. Adv. Anat. Embryol. Cell Biol..

[10] Pannese E. (2002). Perikaryal Surface Specializations of Neurons in Sensory Ganglia. Int. Rev. Cytol..

[11] Miller K.E., Richards B.A., Kriebel R.M. (2002). Glutamine-, Glutamine Synthetase-, Glutamate Dehydrogenase- and Pyruvate Carboxylase-Immunoreactivities in the Rat Dorsal Root Ganglion and Peripheral Nerve. Brain Res..

[12] Lu J., Wang D., Xu J., Zhang H., Yu W. (2023). New Insights on the Role of Satellite Glial Cells. Stem Cell Rev. Rep..

[13] Berger U.V., Hediger M.A. (2000). Distribution of the Glutamate Transporters GLAST and GLT-1 in Rat Circumventricular Organs, Meninges, and Dorsal Root Ganglia. J. Comp. Neurol..

[14] Wei N., Liu Y.-P., Wang R.-R., Zhong Z.-J., Wang X.-L., Yang Y., He T., Zhao S.-J., Wang H., Yu Y.-Q. (2022). Glutamine Maintains Satellite Glial Cells Growth and Survival in Culture. Neurochem. Res..

[15] Vit J.-P., Ohara P.T., Bhargava A., Kelley K., Jasmin L. (2008). Silencing the Kir4.1 Potassium Channel Subunit in Satellite Glial Cells of the Rat Trigeminal Ganglion Results in Pain-like Behavior in the Absence of Nerve Injury. J. Neurosci. Off. J. Soc. Neurosci..

[16] Vit J.-P., Jasmin L., Bhargava A., Ohara P.T. (2006). Satellite Glial Cells in the Trigeminal Ganglion as a Determinant of Orofacial Neuropathic Pain. Neuron Glia Biol..

[17] Procacci P., Magnaghi V., Pannese E. (2008). Perineuronal Satellite Cells in Mouse Spinal Ganglia Express the Gap Junction Protein Connexin43 throughout Life with Decline in Old Age. Brain Res. Bull..

[18] George D., Ahrens P., Lambert S. (2018). Satellite Glial Cells Represent a Population of Developmentally Arrested Schwann Cells. Glia.

[19] Avraham O., Deng P.-Y., Jones S., Kuruvilla R., Semenkovich C.F., Klyachko V.A., Cavalli V. (2020). Satellite Glial Cells Promote Regenerative Growth in Sensory Neurons. Nat. Commun..

[20] van Weperen V.Y.H., Littman R.J., Arneson D.V., Contreras J., Yang X., Ajijola O.A. (2021). Single-Cell Transcriptomic Profiling of Satellite Glial Cells in Stellate Ganglia Reveals Developmental and Functional Axial Dynamics. Glia.

[21] Nguyen H.S., Kang S.J., Kim S., Cha B.H., Park K.-S., Jeong S.-W. (2024). Changes in the Expression of Satellite Glial Cell-Specific Markers during Postnatal Development of Rat Sympathetic Ganglia. Brain Res..

[22] Wang K., Wang S., Chen Y., Wu D., Hu X., Lu Y., Wang L., Bao L., Li C., Zhang X. (2021). Publisher Correction: Single-Cell Transcriptomic Analysis of Somatosensory Neurons Uncovers Temporal Development of Neuropathic Pain. Cell Res..

[23] Renthal W., Tochitsky I., Yang L., Cheng Y.-C., Li E., Kawaguchi R., Geschwind D.H., Woolf C.J. (2020). Transcriptional Reprogramming of Distinct Peripheral Sensory Neuron Subtypes after Axonal Injury. Neuron.

[24] Zhang X., Chen Y., Wang C., Huang L.-Y.M. (2007). Neuronal Somatic ATP Release Triggers Neuron-Satellite Glial Cell Communication in Dorsal Root Ganglia. Proc. Natl. Acad. Sci. USA.

[25] Gu Y., Chen Y., Zhang X., Li G.-W., Wang C., Huang L.-Y.M. (2010). Neuronal Soma-Satellite Glial Cell Interactions in Sensory Ganglia and the Participation of Purinergic Receptors. Neuron Glia Biol..

[26] Chen Z., Huang Q., Song X., Ford N.C., Zhang C., Xu Q., Lay M., He S.-Q., Dong X., Hanani M. (2022). Purinergic Signaling between Neurons and Satellite Glial Cells of Mouse Dorsal Root Ganglia Modulates Neuronal Excitability in Vivo. Pain.

[27] Jia S., Liu J., Chu Y., Liu Q., Mai L., Fan W. (2022). Single-Cell RNA Sequencing Reveals Distinct Transcriptional Features of the Purinergic Signaling in Mouse Trigeminal Ganglion. Front. Mol. Neurosci..

[28] Rajasekhar P., Poole D.P., Liedtke W., Bunnett N.W., Veldhuis N.A. (2015). P2Y1 Receptor Activation of the TRPV4 Ion Channel Enhances Purinergic Signaling in Satellite Glial Cells. J. Biol. Chem..

[29] Chen Y., Zhang X., Wang C., Li G., Gu Y., Huang L.-Y.M. (2008). Activation of P2X7 Receptors in Glial Satellite Cells Reduces Pain through Downregulation of P2X3 Receptors in Nociceptive Neurons. Proc. Natl. Acad. Sci. USA.

[30] Castillo C., Norcini M., Martin Hernandez L.A., Correa G., Blanck T.J.J., Recio-Pinto E. (2013). Satellite Glia Cells in Dorsal Root Ganglia Express Functional NMDA Receptors. Neuroscience.

[31] Castillo C., Norcini M., Baquero-Buitrago J., Levacic D., Medina R., Montoya-Gacharna J.V., Blanck T.J.J., Dubois M., Recio-Pinto E. (2011). The N-Methyl-D-Aspartate-Evoked Cytoplasmic Calcium Increase in Adult Rat Dorsal Root Ganglion Neuronal Somata Was Potentiated by Substance P Pretreatment in a Protein Kinase C-Dependent Manner. Neuroscience.

[32] Kung L.-H., Gong K., Adedoyin M., Ng J., Bhargava A., Ohara P.T., Jasmin L. (2013). Evidence for Glutamate as a Neuroglial Transmitter within Sensory Ganglia. PLoS ONE.

[33] Jeftinija S., Jeftinija K., Liu F., Skilling S.R., Smullin D.H., Larson A.A. (1991). Excitatory Amino Acids Are Released from Rat Primary Afferent Neurons in Vitro. Neurosci. Lett..

[34] Jo H.J., Kim J.S., Kim N.G., Lee K.S., Choi J.H. (2013). Redoable Tie-over Dressing Using Multiple Loop Silk Threads. Arch. Plast. Surg..

[35] Afroz S., Arakaki R., Iwasa T., Oshima M., Hosoki M., Inoue M., Baba O., Okayama Y., Matsuka Y. (2019). CGRP Induces Differential Regulation of Cytokines from Satellite Glial Cells in Trigeminal Ganglia and Orofacial Nociception. Int. J. Mol. Sci..

[36] Leo M., Schmitt L.-I., Kutritz A., Kleinschnitz C., Hagenacker T. (2021). Cisplatin-Induced Activation and Functional Modulation of Satellite Glial Cells Lead to Cytokine-Mediated Modulation of Sensory Neuron Excitability. Exp. Neurol..

[37] Hanani M. (2022). How Is Peripheral Injury Signaled to Satellite Glial Cells in Sensory Ganglia?. Cells.

[38] Hou X.E., Lundmark K., Dahlström A.B. (1998). Cellular Reactions to Axotomy in Rat Superior Cervical Ganglia Includes Apoptotic Cell Death. J. Neurocytol..

[39] Hendry I.A., Campbell J. (1976). Morphometric Analysis of Rat Superior Cervical Ganglion after Axotomy and Nerve Growth Factor Treatment. J. Neurocytol..

[40] Gunjigake K.K., Goto T., Nakao K., Kobayashi S., Yamaguchi K. (2009). Activation of Satellite Glial Cells in Rat Trigeminal Ganglion after Upper Molar Extraction. Acta Histochem. Cytochem..

[41] Donegan M., Kernisant M., Cua C., Jasmin L., Ohara P.T. (2013). Satellite Glial Cell Proliferation in the Trigeminal Ganglia after Chronic Constriction Injury of the Infraorbital Nerve. Glia.

[42] Dixon J.S. (1969). Changes in the Fine Structure of Satellite Cells Surrounding Chromatolytic Neurons. Anat. Rec..

[43] Hu P., McLachlan E.M. (2002). Macrophage and Lymphocyte Invasion of Dorsal Root Ganglia after Peripheral Nerve Lesions in the Rat. Neuroscience.

[44] Jager S.E., Pallesen L.T., Richner M., Harley P., Hore Z., McMahon S., Denk F., Vaegter C.B. (2020). Changes in the Transcriptional Fingerprint of Satellite Glial Cells Following Peripheral Nerve Injury. Glia.

[45] Avraham O., Feng R., Ewan E.E., Rustenhoven J., Zhao G., Cavalli V. (2021). Profiling Sensory Neuron Microenvironment after Peripheral and Central Axon Injury Reveals Key Pathways for Neural Repair. eLife.

[46] van Velzen M., Laman J.D., Kleinjan A., Poot A., Osterhaus A.D.M.E., Verjans G.M.G.M. (2009). Neuron-Interacting Satellite Glial Cells in Human Trigeminal Ganglia Have an APC Phenotype. J. Immunol..

[47] Woodham P., Anderson P.N., Nadim W., Turmaine M. (1989). Satellite Cells Surrounding Axotomised Rat Dorsal Root Ganglion Cells Increase Expression of a GFAP-like Protein. Neurosci. Lett..

[48] Liu H., Zhao L., Gu W., Liu Q., Gao Z., Zhu X., Wu Z., He H., Huang F., Fan W. (2018). Activation of Satellite Glial Cells in Trigeminal Ganglion Following Dental Injury and Inflammation. J. Mol. Histol..

[49] Konnova E.A., Deftu A.-F., Chu Sin Chung P., Pertin M., Kirschmann G., Decosterd I., Suter M.R. (2023). Characterisation of GFAP-Expressing Glial Cells in the Dorsal Root Ganglion after Spared Nerve Injury. Int. J. Mol. Sci..

[50] Sullivan S.M., Lee A., Björkman S.T., Miller S.M., Sullivan R.K.P., Poronnik P., Colditz P.B., Pow D.V. (2007). Cytoskeletal Anchoring of GLAST Determines Susceptibility to Brain Damage: An Identified Role for GFAP. J. Biol. Chem..

[51] Mohr K.M., Pallesen L.T., Richner M., Vaegter C.B. (2021). Discrepancy in the Usage of GFAP as a Marker of Satellite Glial Cell Reactivity. Biomedicines.

[52] Ohara P.T., Vit J.-P., Bhargava A., Jasmin L. (2008). Evidence for a Role of Connexin 43 in Trigeminal Pain Using RNA Interference in Vivo. J. Neurophysiol..

[53] Kaji K., Shinoda M., Honda K., Unno S., Shimizu N., Iwata K. (2016). Connexin 43 Contributes to Ectopic Orofacial Pain Following Inferior Alveolar Nerve Injury. Mol. Pain.

[54] Zhang H., Mei X., Zhang P., Ma C., White F.A., Donnelly D.F., Lamotte R.H. (2009). Altered Functional Properties of Satellite Glial Cells in Compressed Spinal Ganglia. Glia.

[55] Ji R.-R., Chamessian A., Zhang Y.-Q. (2016). Pain Regulation by Non-Neuronal Cells and Inflammation. Science.

[56] Xu J.-T., Xin W.-J., Zang Y., Wu C.-Y., Liu X.-G. (2006). The Role of Tumor Necrosis Factor-Alpha in the Neuropathic Pain Induced by Lumbar 5 Ventral Root Transection in Rat. Pain.

[57] Ohtori S., Takahashi K., Moriya H., Myers R.R. (2004). TNF-Alpha and TNF-Alpha Receptor Type 1 Upregulation in Glia and Neurons after Peripheral Nerve Injury: Studies in Murine DRG and Spinal Cord. Spine.

[58] Dubový P., Klusáková I., Svízenská I., Brázda V. (2010). Satellite Glial Cells Express IL-6 and Corresponding Signal-Transducing Receptors in the Dorsal Root Ganglia of Rat Neuropathic Pain Model. Neuron Glia Biol..

[59] Dubový P., Brázda V., Klusáková I., Hradilová-Svíženská I. (2013). Bilateral Elevation of Interleukin-6 Protein and mRNA in Both Lumbar and Cervical Dorsal Root Ganglia Following Unilateral Chronic Compression Injury of the Sciatic Nerve. J. Neuroinflamm..

[60] Zhou X.F., Deng Y.S., Chie E., Xue Q., Zhong J.H., McLachlan E.M., Rush R.A., Xian C.J. (1999). Satellite-Cell-Derived Nerve Growth Factor and Neurotrophin-3 Are Involved in Noradrenergic Sprouting in the Dorsal Root Ganglia Following Peripheral Nerve Injury in the Rat. Eur. J. Neurosci..

[61] Grothe C., Meisinger C., Hertenstein A., Kurz H., Wewetzer K. (1997). Expression of Fibroblast Growth Factor-2 and Fibroblast Growth Factor Receptor 1 Messenger RNAs in Spinal Ganglia and Sciatic Nerve: Regulation after Peripheral Nerve Lesion. Neuroscience.

[62] Grothe C., Meisinger C., Claus P. (2001). In Vivo Expression and Localization of the Fibroblast Growth Factor System in the Intact and Lesioned Rat Peripheral Nerve and Spinal Ganglia. J. Comp. Neurol..

[63] Klimaschewski L., Meisinger C., Grothe C. (1999). Localization and Regulation of Basic Fibroblast Growth Factor (FGF-2) and FGF Receptor-1 in Rat Superior Cervical Ganglion after Axotomy. J. Neurobiol..

[64] Xian C.J., Zhou X.F. (1999). Neuronal-Glial Differential Expression of TGF-Alpha and Its Receptor in the Dorsal Root Ganglia in Response to Sciatic Nerve Lesion. Exp. Neurol..

[65] McGinnis A., Ji R.-R. (2023). The Similar and Distinct Roles of Satellite Glial Cells and Spinal Astrocytes in Neuropathic Pain. Cells.

[66] Huang L.-Y.M., Gu Y., Chen Y. (2013). Communication between Neuronal Somata and Satellite Glial Cells in Sensory Ganglia. Glia.

[67] Leibinger M., Müller A., Gobrecht P., Diekmann H., Andreadaki A., Fischer D. (2013). Interleukin-6 Contributes to CNS Axon Regeneration upon Inflammatory Stimulation. Cell Death Dis..

[68] Zhao Q., Jiang C., Zhao L., Dai X., Yi S. (2024). Unleashing Axonal Regeneration Capacities: Neuronal and Non-Neuronal Changes After Injuries to Dorsal Root Ganglion Neuron Central and Peripheral Axonal Branches. Mol. Neurobiol..

[69] Brown R.I., Barber H.M., Kucenas S. (2024). Satellite Glial Cell Manipulation Prior to Axotomy Enhances Developing Dorsal Root Ganglion Central Branch Regrowth into the Spinal Cord. Glia.

[70] Ruan Y., Cheng J., Dai J., Ma Z., Luo S., Yan R., Wang L., Zhou J., Yu B., Tong X. (2023). Chronic Stress Hinders Sensory Axon Regeneration via Impairing Mitochondrial Cristae and OXPHOS. Sci. Adv..

[71] Hervera A., De Virgiliis F., Palmisano I., Zhou L., Tantardini E., Kong G., Hutson T., Danzi M.C., Perry R.B.-T., Santos C.X.C. (2018). Reactive Oxygen Species Regulate Axonal Regeneration through the Release of Exosomal NADPH Oxidase 2 Complexes into Injured Axons. Nat. Cell Biol..

[72] Schulte A., Lohner H., Degenbeck J., Segebarth D., Rittner H.L., Blum R., Aue A. (2023). Unbiased Analysis of the Dorsal Root Ganglion after Peripheral Nerve Injury: No Neuronal Loss, No Gliosis, but Satellite Glial Cell Plasticity. Pain.

